# 《胸部恶性肿瘤围术期静脉血栓栓塞症预防中国专家共识（2018版）》解读之围术期高凝状态篇

**DOI:** 10.3779/j.issn.1009-3419.2019.12.03

**Published:** 2019-12-20

**Authors:** 松平 崔, 辉 李

**Affiliations:** 北京，首都医科大学附属北京朝阳医院胸外科 Department of Thoracic Surgery, Beijing Chaoyang Hospital Affiliated to Capital Medical University, Beijing 100020, China

**Keywords:** 肺肿瘤, 静脉血栓栓塞症, 高凝状态, 生物标记物, Lung neoplasms, Venous thromboembolism, Hypercoagulable state, Biomarker

## Abstract

静脉血栓栓塞症（venous thromboembolism, VTE）是肺癌围术期常见的并发症，也是导致院内非预期死亡的重要原因。VTE临床相关的高危因素包括：患者自身的因素（高龄、肥胖等）、肿瘤相关因素（分型、分期等）、治疗相关因素（化疗、手术等）。除此之外，肿瘤细胞会表达癌性促凝因子（cancer procoagulant, CP）、组织因子（tissue factor, TF）、炎症因子等，或是激活血小板、炎性细胞等相关细胞，直接或间接地活化凝血过程，引起血液高凝状态，从而促进VTE的发生。同时，相关的生物标记物也可反映肺癌患者围术期的凝血状态，这有助于更准确地筛选VTE高危患者，更精准地予以预防策略。

## Abstract (en)

Venous thromboembolism (VTE) is a common perioperative complication of lung cancer and a major cause of unexpected death in hospital. The clinical risk factors of VTE include: patients' factors (advanced age, obesity, *etc.*), tumor-related factors (classification, staging, *etc.*), treatment-related factors (chemotherapy, surgery, *etc.*). In addition, tumor cells express cancer procoagulant (CP), tissue factor (TF), inflammatory factors or activate platelets, inflammatory cells and other related cells, directly or indirectly activate the coagulation process, and cause blood hypercoagulable state, thus promote the occurrence of VTE. At the same time, the relevant biomarkers can also reflect the perioperative coagulation status of patients, which is helpful to more accurately identify high-risk subgroups to establish more accurate and targeted anticoagulation strategies to prevent thrombosis in lung cancer patients.

随着人们生活环境以及生活方式的变化，尤其是吸烟人数的不断增长，肺癌的发病率和死亡率日益增加，严重危害着人们的健康和生命。目前，外科手术仍是肺癌首选的治疗方式，而静脉血栓栓塞症（venous thromboembolism, VTE）是肺癌手术围术期的常见并发症，也是癌症患者的第二大死亡原因^[1]^。

早在150多年前，Trousseau提出了癌症患者常合并VTE，并指出VTE是隐匿性恶性肿瘤的早期表现^[2]^。一项关于肺手术患者入院时VTE发生率的研究^[3]^显示：肺癌患者入院时VTE发生率为5.2%（12/231），肺良性肿瘤患者入院时未见VTE发生（0/77），两者具有统计学差异。另一项关于肺术后患者新发VTE的研究^[4]^显示：肺术后VTE发生率为11.5%，其中，良性肿瘤术后VTE发生率为7.0%，恶性肿瘤术后VTE发生率为15.0%。这也意味着，肺癌患者术前及术后VTE发生率均高于肺良性肿瘤患者，这与肺癌相关的血液高凝状态（hypercoagulable state）密切相关^[5]^。

高凝状态是指多种因素引起的止血、凝血、抗凝和纤溶等功能紊乱，进而促进血栓形成的病理状态。高凝状态的形成是一个极其复杂的过程，多种因素的相互作用在疾病的发展中共同激活了凝血级联反应，主要包括患者相关因素（高龄、肥胖、糖尿病等）、肿瘤相关因素（类型、分期、局部浸润、淋巴结转移、远处转移等）以及抗肿瘤治疗相关因素（化疗、靶向治疗、手术等）。肿瘤相关的促凝机制在VTE的发生、发展中起着至关重要的作用，这其中包括释放促凝因子、调控正常细胞、表达表面黏附分子等。《胸部恶性肿瘤围术期静脉血栓栓塞症预防中国专家共识（2018版）》（以下简称《共识》）已对临床相关因素进行了详细叙述，遂本文就肿瘤细胞相关促凝机制展开综述，明确肺癌围术期高凝状态相关因素，为进一步筛选VTE高危人群进行预防性抗凝提供参考。

## 1 肿瘤细胞相关促凝机制

越来越多的研究表明肿瘤细胞通过表达癌性促凝因子（cancer procoagulant, CP）、组织因子（tissue factor, TF）、炎症因子[肿瘤坏死因子α（tumor necrosis factor-α, TNF-α）、白介素1（interleukin-1, IL-1）等]，表达细胞间黏附分子（cell adhesion molecules, I-CAM）以及激活血小板、炎性细胞等方式，直接或间接地激活凝血过程，造成血液高凝状态，从而促进VTE的发生。

### 1.1 直接促凝机制

肿瘤细胞的直接促凝机制包括表达促凝因子以及细胞表面黏附分子。促凝因子中，最重要的为TF和CP。

TF是一种跨膜蛋白，是外源性凝血途径的始动因子，通过与凝血因子Ⅶ结合成复合物，激活外源性凝血途径，最终使凝血酶原转化为凝血酶。许多肿瘤细胞会持续高表达TF，TF水平与肿瘤浸润、转移、血管新生、血栓形成有着明确的相关性^[6-8]^。与此同时，肿瘤细胞会持续表达细胞外囊泡（extracellular vesicles, EVs），这也是循环中TF的主要来源^[9]^。TF+EVs不仅能激活外源性凝血途径，还可以激活血管内皮细胞，形成高凝状态^[10]^。近期相关研究^[11]^显示多种肿瘤组织中TF的高表达与预后不良相关，其中包括胰腺癌、胃癌、直肠癌、乳腺癌等，但肺癌相关研究较少。

CP是一种维生素K依赖蛋白质，可不依赖Ⅶ因子，通过直接激活凝血因子X参与凝血过程。通常，CP在正常细胞呈阴性表达，但在肺癌、肾癌、乳腺癌、结肠癌以及血液系统肿瘤细胞中呈高表达，进而引起血液高凝状态。

ICAM-1属于免疫球蛋白超家族，是一种被重度糖基化修饰的单跨膜单链糖蛋白。肿瘤细胞可通过高表达ICAM-1增加细胞黏附性，从而增加与内皮细胞间的连接，促进局部血液凝结，促进肿瘤浸润、转移；肿瘤细胞表面的ICAM-1可自行或在酶的作用下脱落形成血清可溶性细胞间黏附分子1（soluble intercellular adhesion molecule-1, sICAM-1），其包含ICAM-1绝大部分胞外域，在体液循环中以二聚体或多聚体的形式存在，这种结构可增加与细胞功能相关抗原1（lymphocyte function associated antigen-1, LFA-1）的结合能力^[12, 13]^，从而抑制循环细胞毒性T淋巴细胞，协助肿瘤细胞逃过自身免疫识别，更易侵袭和转移^[14, 15]^。

### 1.2 间接促凝机制

肿瘤的间接促凝机制是通过合成、释放可溶性调控因子或黏附激活机体正常细胞（内皮细胞、血小板、白细胞等），使其发生相应的促凝改变，促进血栓的形成。其中调控因子包括炎症因子（TNF-α、IL-1β等）、促血管新生因子[血管内皮生长因子（vascular endothelial growth factor, VEGF）、粒细胞集落刺激因子（granulocyte colony stimulating factor, G-CSF）等]以及血小板聚集激动因子等。

TNF-α、IL-1β等炎症因子的高表达可显著增加肿瘤患者发生VTE的风险^[16-19]^。因其可促进血管内皮细胞及单核细胞高表达TF并下调血栓调节蛋白（thrombomodulin, TM）的表达，而TM可与凝血酶结合激活蛋白C抗凝通路，其表达的下调可导致血液的高凝状态。除此之外，TNF-α、IL-1β还可刺激内皮细胞产生纤溶酶抑制剂，抑制血栓的溶解，使血栓更加牢固。

VEGF是一种多功能糖基化多肽因子，通过刺激内皮细胞增殖，促进新生血管的形成和新生血管网的建立，是最强、最特异的肿瘤血管生成调节因子。此外，VEGF也可通过改变血管内皮结构的完整性来调节血管通透性，从而更易引起血栓形成。肺癌患者TF和VEGF均呈高表达状态，TF也可通过刺激内皮细胞表达VEGF，这使得肺癌患者血液中VEGF浓度较高，促进了新生血管以及高凝状态的形成^[20]^。

G-CSF属于生长因子家族，主要由血管内皮细胞、单核巨噬细胞、成纤维细胞和骨髓间质细胞产生，能够促进中性粒细胞生长和激活。癌症患者循环血液中的G-CSF水平常升高，并且与不良预后相关^[21]^。被激活的中性粒细胞可释放中性粒细胞外陷阱（neutrophil extracellular traps, NETs），而NETs可诱导血小板的聚集、凝血酶的激活和纤维蛋白多聚体的形成，从而导致血管内皮损伤及血栓形成。NETs在肿瘤患者中的高表达是肿瘤相关高凝状态的重要因素，同时也可能为肿瘤相关VTE的预防和治疗提供了新的靶点^[22-24]^。

肿瘤细胞还可释放血小板聚集和活化介质，包括二磷酸腺苷、血栓素A2（thromboxane A2, TXA2）或肿瘤相关蛋白等，而活化的血小板可释放VEGF等多种促血管生成因子，影响血管内皮的完整性^[25]^，并增加肿瘤细胞对血管内皮的黏附作用，这是血栓形成的重要机制，也是肿瘤血行转移的重要因素^[26, 27]^。

肿瘤细胞也可以刺激内皮细胞表达的多种表面黏附分子，引起局部凝血的激活及血栓的形成，促进肿瘤的侵袭、转移，其中包括选择素、整合素、免疫球蛋白等。P-选择素是一种很重要的细胞黏附分子，可由被激活的内皮细胞和活化的血小板表达，介导活化血小板与肿瘤细胞的聚集以及肿瘤细胞黏附于内皮细胞。此外，P-选择素还可介导EVs在细胞之间的转运，进一步促进微血栓的形成^[28]^。肿瘤细胞、血小板、白细胞与血管内皮细胞的黏附有利于促凝因子、肿瘤相关调控因子、EVs释放于血管内皮，这也构成了高凝状态和微血栓形成的基础。

## 2 VTE及高凝状态评估

临床工作中，我们常根据患者所存在的高危因素，采用风险评估模型进行VTE风险分层，从而针对性地予以预防措施。目前，常用的风险评估模型有Caprini风险评估量表（Caprini risk assessment model, Caprini RAM）^[29]^、Padua评分量表^[30]^、Rogers评分量表^[31]^和Khorana评分量表^[32]^。目前外科常用的是Caprini RAM，其中涵盖了40多个住院患者VTE相关因素，如年龄、下肢静脉曲张、肺功能异常等。近些年来，一种改良简化的Caprini RAM已被国外胸外科医师所应用。改良的Caprini RAM简化了经典量表内的相关因素，并将VTE风险等级分为低危（0分-4分）、中危（5分-8分）和高危（≥9分），其在胸部恶性肿瘤患者VTE风险评估中有着更优的信效度^[33-35]^。因此，《共识》也推荐应用改良Caprini RAM进行胸部恶性肿瘤患者VTE风险动态评估。

高凝状态是多种因素引起凝血-抗凝平衡的紊乱，因此常规的凝血检查[凝血酶原时间（prothrombin time, PT）、活化部分凝血活酶时间（activated partial thromboplastin time, APTT）等]在高凝状态评估中具有一定的局限性^[36]^。而血栓弹力图（thromboelastography, TEG）是通过在体外模拟血栓形成的过程，展现凝血、抗凝、纤溶等凝血过程的全貌，现已被广泛用于凝血状态的评估以及预防性抗凝的监测^[37-39]^。TEG参数主要包括R、K、Angel、MA、G、CI等，其中CI是由R、K、MA、Angel计算得来，而非直接测定，CI可以综合评估凝血状态。根据病因将高凝状态分为3类：①酶相关高凝状态，CI > 3，R≤5 min，MA≤70 mm；②血小板相关高凝状态，CI > 3，R > 5 min，MA > 70 mm；③复合高凝状态，CI > 3，R≤5 min，MA > 70 mm^[40]^。也有相关研究指出具有R降低、Angle增大、MA升高其中两项^[36]^或者G≥11 dynes/cm²^[38]^亦可诊断为高凝状态。在胸外科方面有研究^[41, 42]^显示，肺癌围术期并不存在高凝状态，且预防性抗凝并不能改变患者的凝血状态。但相关研究样本量较小，仍需大样本临床实验验证。

## 3 总结

肺癌围术期VTE发生率较高，这与肺癌患者自身血液高凝状态密切相关。因此，充分地认识高凝状态、精准地识别高危人群、个体化地应用预防性抗凝以及规范地进行抗凝监测是肺癌围术期VTE管理必不可少的部分。无论是经典Caprini RAM还是改良的Caprini RAM，多着眼于患者的临床特征，对于凝血相关的实验室指标均无涉及，D二聚体、血小板计数、P-选择素、TF等实验室指标与围术期VTE的发生密切相关，对于VTE的预测有着重要意义。未来，临床特征与实验室检查相结合的VTE风险评估量表会更准确地识别VTE高危人群，从而针对性地应用预防性抗凝药物，降低VTE发生率的同时优化抗凝的风险-收益比率。对于肺癌患者围术期高凝状态的认知，很可能会给予临床医生更多的策略进行VTE的防治。
